# Comorbidities and Consequences in Hospitalized Heart Failure Patients with Depression

**DOI:** 10.7759/cureus.3193

**Published:** 2018-08-23

**Authors:** Rikinkumar S Patel, Shristi Shrestha, Hina Saeed, Sanjeetha Raveendranathan, Ehinor E Isidahome, Virendrasinh Ravat, Mary O Fakorede, Viralkumar Patel

**Affiliations:** 1 Department of Psychiatry, Griffin Memorial Hospital, Norman, USA; 2 Public Health, State University of New York, Albany, USA; 3 Psychiatry, Sindh Medical, Ontario , CAN; 4 Medicine, SABA University School of Medicine, Devens, MA, USA; 5 Department of Internal Medicine, Saint Joseph Hospital, London, USA; 6 Department of Infectious Disease, Clinical Infectious Disease Specialist, Las Vegas, USA; 7 Psychiatry, Ladoke Akintola University, Ogbomoso, NGA; 8 Internal Medicine, Blake Medical Center, Bradenton, USA

**Keywords:** major depressive disorder, mortality risk, heart failure, depression, comorbidities, demographics, concomitant hypothyroidism, hospitalization cost, hospital stay, healthcare cost and utilization project (hcup)

## Abstract

Objective

To evaluate the demographic predictors of major depressive disorder (MDD) in hospitalized congestive heart failure (CHF) patients and measure the differences in hospital stay and cost per comorbidities and the associated risk of in-hospital mortality.

Methods

This retrospective cross-sectional study used nationwide inpatient data from the healthcare cost and utilization project (HCUP). We identified patients with CHF as the primary diagnosis and MDD as the secondary diagnosis using ICD-9-CM codes and compared with the CHF patient without MDD. The differences in comorbidities were quantified using chi-square tests and the logistic regression model was used to evaluate mortality risk among comorbidities using odds ratio (OR).

Results

Elder CHF patients, 36–50-year-old (OR: 1.324) and whites (OR: 1.673), have a higher likelihood of a co-diagnosis of MDD. Females with heart failure have two-fold higher odds of MDD (OR: 2.332). Majority of the medical comorbidities were seen in a higher proportion of CHF patients without MDD. Hypothyroidism (10.2%) and drug abuse (15.2%) were seen more in depressed patients comparatively. Among substance use disorder, patients with drug abuse stayed longer and had a higher hospitalization total cost ($51,828). And, hypothyroidism was associated with longer inpatient stay (5.6 days) and cost ($64,726), and four-fold higher odds of in-hospital mortality (OR: 4.405). Though alcohol abuse was seen only in 7.4% of CHF patients with MDD, it was associated with the three-fold higher likelihood of deaths during hospitalization (OR: 3.195).

Conclusion

A middle-aged, white female with comorbid depression has a higher risk of hospitalization for heart failure. Depressed CHF patients with comorbid hypothyroidism were hospitalized for a longer duration with higher inpatient cost and four times higher risk of mortality during hospitalization stay. Further studies are required to evaluate the underlying cause of worse hospital outcomes in depressed CHF patients with alcohol abuse and hypothyroidism. An integrated healthcare model is required for early diagnosis and treatment of depression and associated comorbidities in CHF patients to reduce mortality and improve post-CHF outcomes.

## Introduction

About 4.8 million Americans are suffering from congestive heart failure (CHF) and around 400,000 incidences report each year as per the National Heart, Lung and Blood Institute [1]. A study analyzing the prevalence rates of depression in heart failure patients showed that there is an estimation of 27.8% and higher prevalence linked with increased New York Heart Association (NYHA) class [2]. Several biological pathways have been linked with both depression and heart failure that seem to be interlinked. Both are related to an activated sympathetic system and increased hormones such as the interleukins (IL6, IL1B), tumor necrosis factor-alpha (TNF-alpha), etc. [2]. It has been postulated that the inflammatory process associated with depression can further exacerbate the pathophysiologic process that deteriorates the condition of heart failure patients [2].

Approximately half of CHF patients with the major depressive disorder (MDD) have clinical depressive symptoms of high severity [3]. Several prospective studies, systematic and meta-analytic reviews have deductively concluded a staggeringly two-fold risk in all-cause mortality and morbidity rates amongst the CHF patients with comorbid depressive symptomatology [4]. The largest comprehensive hospital-based registry analysis conducted to date, Organized Program to Initiate Lifesaving Treatment in Hospitalized Patients with Heart Failure (OPTIMIZE-HF) pooled data from 48,600 hospitalized CHF patients, states that the quality of life and clinical outcome of those simultaneously affected with MDD, to be staggeringly poor [5]. Specifically, the CHF cohort with MDD has a short-term mortality risk of 17.7% ensuing within three months of hospital discharge; a risk significantly heightened in comparison to 6.6% in failure only patients [5]. Patients with comorbid depression also tend to have on the average prolonged duration of in-hospital stays (seven days versus four to five days) in addition to a three-fold increased risk of late re-hospitalization, owing to decompensated states with exacerbation of symptoms [6]. Additionally, CHF patients with comorbid MDD tend to have a poor quality of life, higher rate of cardiac-related morbidity, and premature mortality [7]. Comorbid drug and alcohol abuse are poor prognostic factors in a patient with depression and CHF. Studies suggest that alcohol consumption has adverse impacts on the utilization of health care services as well as mental health state in patients with chronic medical disorders and depression [8].

The objective of this nationwide retrospective study is to evaluate the demographic predictors of MDD in hospitalized CHF patients and discern the differences in the prevalence of medical comorbidities and substance use disorders between MDD and non-MDD heart failure patients. Finally, this study will evaluate the length of hospital stay and cost per comorbidities and the associated risk of in-hospital mortality in CHF patients with MDD.

## Materials and methods

Data source

A retrospective cross-sectional analysis was conducted using the healthcare cost and utilization project's (HCUP) nationwide inpatient sample (NIS) data from the years 2010 to 2014 [9]. The agency for healthcare research and quality (AHRQ) sponsors the HCUP databases that are specifically designed to determine and identify patterns in hospital utilization and cost across the United States [9]. The NIS is the largest inpatient database available in the US, with a sample estimate of over 95% of discharges from the non-federal community hospitals. To protect the privacy of patients, physicians, and hospitals, the state and hospital identifiers are de-identified.

Sample selection

Based on the International Classification of Diseases, Ninth Revision, Clinical Modification (ICD-9-CM) diagnosis codes, we identified the target group as the patients admitted in the hospital with a primary diagnosis of CHF and secondary diagnosis as MDD, and compared with the patients without MDD (comparison group). In HCUP databases, more than 14,000 ICD-9-CM diagnosis codes and 3,900 procedure codes had been mentioned. CHF was identified using diagnosis code 398.91, 428.0, 428.1, 428.20–428.23, 428.30–428.33, 428.40–428.43 or 428.9, and MDD was identified using diagnosis codes 296.20–296.26 or 296.30–296.36. The comparison group was matched with the target group on the basis of age, race, and sex.

Variables of interest

Demographic variables examined in this study included age group (18–35, 36–50), gender (male or female), race (white, non-white) and median household income (above or below 50th percentile). We calculated the length of inpatient stay as the number of nights the patient was hospitalized for a primary diagnosis of CHF. Total charges of hospitalization do not include professional fees and non-covered charges. If the source provided total charges with professional fees, then such additional fees were eliminated from the charge during HCUP data processing. In the NIS, we defined death as in-hospital mortality, and in this study, it is described as all-cause. Comorbidities are considered coexisting conditions to CHF, which is the primary disorder under this study [9]. Based on existing literature, we identified comorbidities for CHF using ICD-9-CM diagnosis codes as shown in Table 1.

**Table 1:** ICD-9-CM diagnosis codes for comorbidities. ICD-9-CM: International Classification of Diseases, 9th Revision, Clinical Modification

Comorbidity	ICD-9-CM diagnosis codes
Anemia	280.1-281.9, 285.21-285.29, 285.9
Chronic lung disease	490-492.8, 493.00-493.92, 494-494.1, 495.0-505, 506.4
Diabetes	249.00-249.31, 250.00-250.33, 648.00-648.04
Hypertension	401.1, 401.9, 642.00-642.04, 401.0, 402.00-405.99, 437.2, 642.10-642.24, 642.70-642.94
Obesity	278.0, 278.00, 278.01, 278.03, 649.10-649.14, 793.91, V85.30-V85.39, V85.41-V85.45, V85.54
Hypothyroidism	243-244.2, 244.8, 244.9
Drug abuse	292.0, 292.82-292.89, 292.9, 304.00-304.93, 305.20-305.93, 648.30-648.34
Alcohol abuse	291.0-291.3, 291.5, 291.8, 291.81, 281.82, 291.89, 291.9, 303.00-303.93, 305.00-305.03

Approaches

Descriptive statistics and cross tabulation were used to summarize the distribution between CHF patients with versus without MDD in terms of demographic characteristics and comorbidities. Pearson’s chi-square test and independent sample T-test was used for categorical data and continuous data respectively to generate the P values between both the groups. We used a logistic regression model to measure the demographic predictors and the risk of in-hospital mortality in CHF patients with MDD using the comparison group as the reference category. The odds ratio (OR) generated by the regression model for in-hospital mortality risk was adjusted for demographics (age, race, sex and median household income). We used discharge weight, which is given in the NIS, to obtain nationally representative inpatient data [9]. Based upon the sample size, a P value < .01 was used as a reference to determine the statistical significance of the test. Statistical analysis was performed using SPSS version 23 (IBM, Armonk, New York, USA) in this study. NIS used in this study does not contain patient identification and so we were not required to take Institution Review Board permission.

## Results

Demographic predictors of MDD

Elder CHF patients (36–50-year-old) have a higher likelihood of a co-diagnosis of MDD (OR: 1.324). Females with heart failure have two-fold higher odds of MDD (OR: 2.332) as there were 32.1% females without MDD versus 52.5% with MDD. MDD was seen in the nearly equal proportion of whites and non-white population (48% and 52%, respectively). But, when compared with patients without MDD then whites were more likely to have MDD (OR: 1.673) associated with a primary diagnosis of CHF. Also, heart failure patients with median household income below the 50th percentile were at a higher risk of depression (OR: 1.314). Demographic distribution in heart failure patients with/without MDD is shown in Table 2.

**Table 2:** Demographic predictors of depression in hospitalized heart failure patients. The proportion between MDD (-) and MDD (+) was obtained using cross tabulation. Odds ratio and P values were generated using logistic regression model. MDD: Major depressive disorder; OR: Odds ratio; CI: Confidence interval.

Variable	MDD (–)		MDD (+)		Logistic regression model		
	N	%	N	%	OR	95% CI	P-value
Age at time of admission							
18–35 years	1520	15.8	2297	19.9	referent		
36–50 years	8100	84.2	9248	80.1	1.324	1.232–1.421	<.001
Sex							
Male	6530	67.9	5489	47.5	referent		
Female	3090	32.1	6056	52.5	2.332	2.204–2.467	<.001
Race							
Non-white	6195	64.4	5998	52.0	referent		
White	3425	35.6	5547	48.0	1.673	1.583–1.768	<.001
Median household income							
Below 50^th^ percentile	5520	60.3	7468	66.6	1.314	1.241–1.392	<.001
Above 50^th^ percentile	3630	39.7	3738	33.4	referent		

Comorbidities in heart failure patients

**Figure 1:**
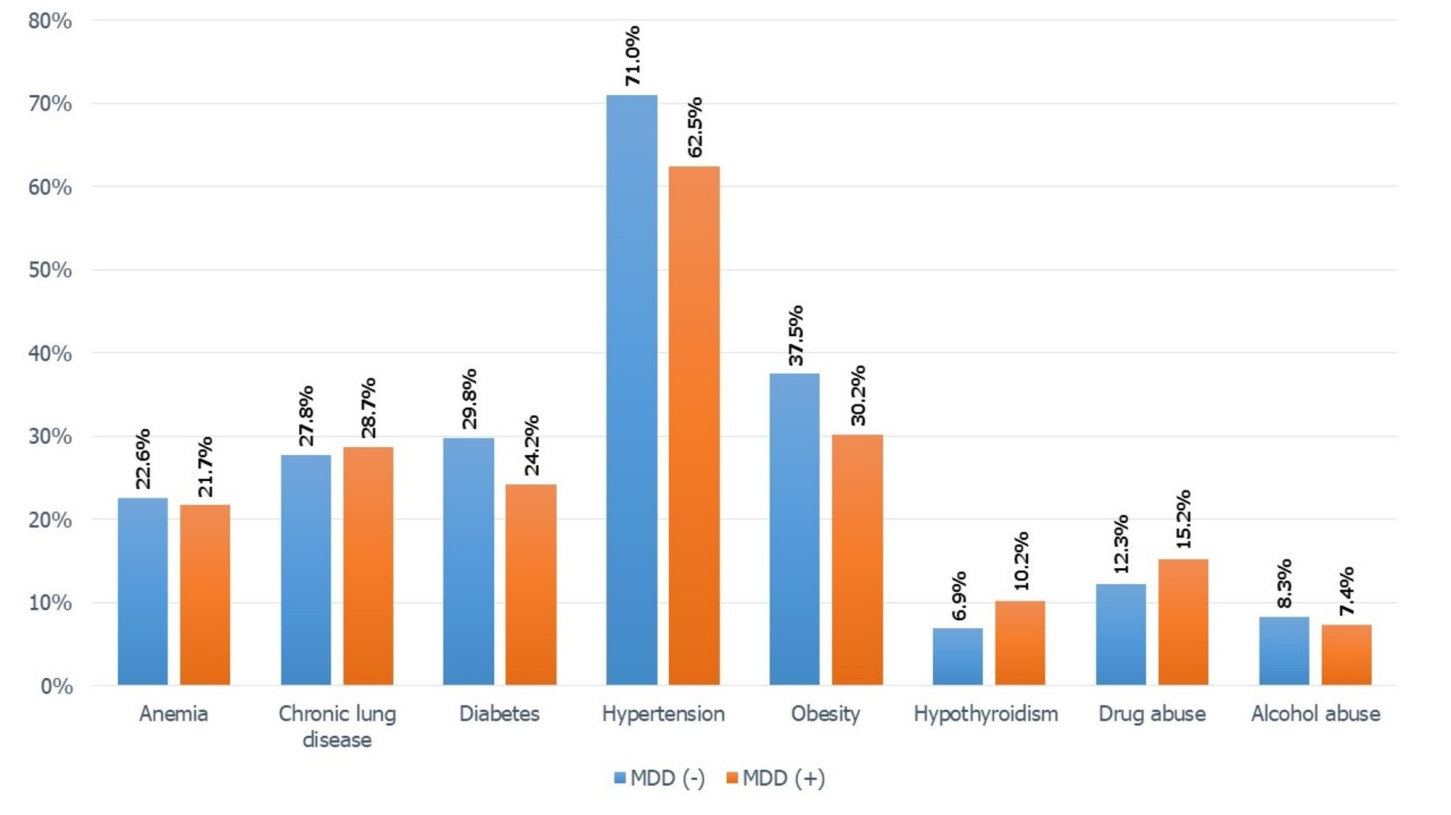
Distribution of comorbidities in heart failure patients with/without MDD. The proportions between MDD (-) and MDD (+) were obtained using cross tabulation. Significant P values ≤ .001 at 95% confidence interval were obtained using the Pearson chi-square (χ2) test. MDD: Major depressive disorder

Majority of the medical comorbidities were seen in the higher proportion of CHF patients without MDD, except hypothyroidism which was present in 10.2% patients with MDD (vs. 6.9% patients without MDD). Also, drug abuse was seen in the higher proportion of depressed patients as shown in Figure 1.

Inpatient stay and cost by comorbidities in MDD patients

**Figure 2:**
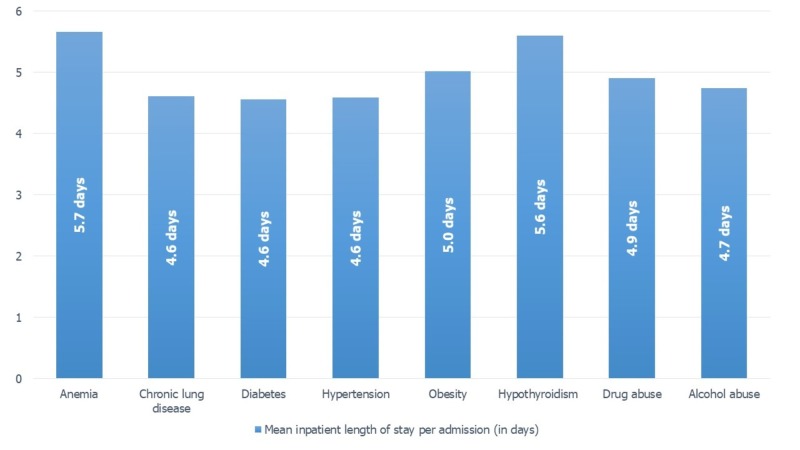
Mean inpatient stay in MDD patients per comorbidities. The mean inpatient length of stay in MDD (+) was obtained using the independent sample T-test. MDD: Major depressive disorder

**Figure 3:**
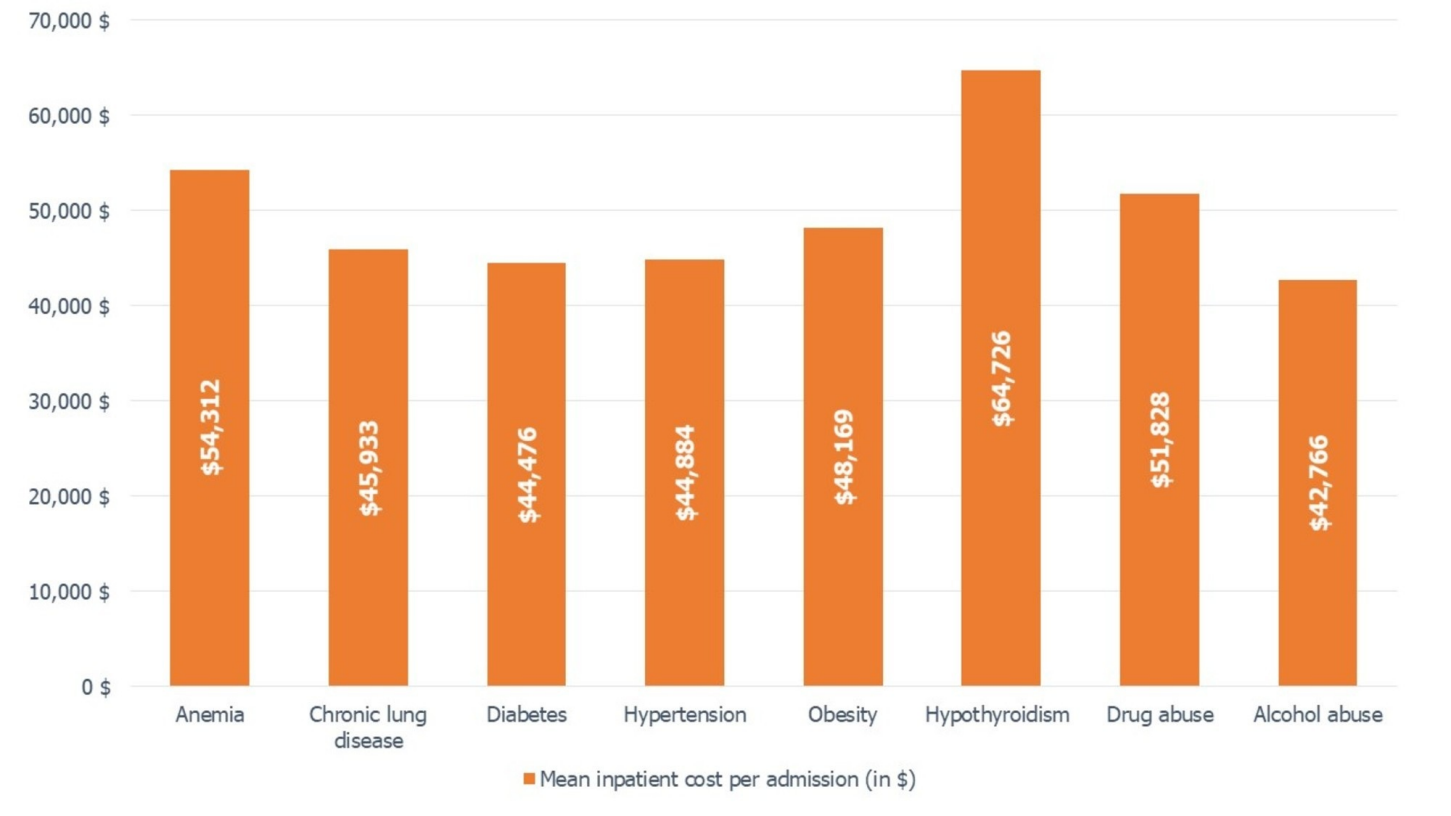
Mean inpatient cost in MDD patients per comorbidities. The mean inpatient cost in MDD (+) was obtained using the independent sample T-test. MDD: Major depressive disorder

Hospitalization stay and cost was highest in MDD patients with comorbid hypothyroidism and anemia as shown in Figure 2 and Figure 3. Among cardiometabolic comorbidities, the length of stay and cost per admission for patients with comorbid diabetes and hypertension was nearly similar. But it was higher for obese patients who stayed for an average of five days and $48,169 per hospital admission for heart failure. Among substance use disorder, patients with co-diagnosis of drug abuse had a higher hospitalization total cost.

Association of comorbidities and mortality in MDD patients

Among the medical comorbidities, hypothyroidism was only associated with higher odds of in-hospital mortality in heart failure patients with MDD (OR: 4.405). Though alcohol abuse was seen only in 7.4% of CHF patients with MDD, it was associated with the three-fold higher likelihood of deaths during hospitalization (OR: 3.195). The association of comorbidities with mortality in MDD patients is shown in Table 3.

**Table 3:** Odds of mortality in heart failure patients with MDD. Odds ratio and P values were generated using logistic regression model and were adjusted for age, gender, race and median household income. Reference category for this model are the patients who did not die. MDD: Major depressive disorder; OR: Odds ratio; CI: Confidence interval.

Comorbidity	OR	95% CI	P-value
Anemia	1.156	.627–2.134	.642
Chronic lung disease	1.191	.651–2.180	.570
Diabetes	.396	.156–1.005	.051
Hypertension	.627	.358–1.097	.102
Obesity	<.001	<.0001	.981
Hypothyroidism	4.405	2.329–8.333	<.001
Drug abuse	.523	.207–1.320	.170
Alcohol abuse	3.195	1.559–6.548	.002

## Discussion

In our study, with four-fifths of the total heart failure patients with MDD in 36–50 years’ age group, the elder patients had a higher risk compared to young adults for comorbid depression. Also, females with CHF had a twice higher risk of comorbid MDD compared to men, and this was supported by a past study which concluded that the average prevalence rate of depression in CHF patients for women was higher than that for men [2]. Whites had a higher risk of depression compared to non-white races.

Both anaemia and depression are significantly prevalent in CHF as independent comorbidities [10, 11]. In addition to similar presentation and overlap of symptoms, studies have also shown an increased prevalence of anaemia in depressed patients [12]. However, limited data are available on the coexistence of these two disorders in CHF and pathophysiology of the possible linkage needs to be explored. In our study, the nearly marginally lower proportion of patients with MDD had anaemia compared to non-MDD group (21.7% vs. 22.6%). Also, we found that the hospitalization stays and cost for heart failure management in patients with depression and comorbid anaemia was much higher compared to other comorbidities. Past studies suggest earlier mortality in patients suffering from depression and this observation has been linked to medical disorders including diabetes and vascular diseases in these subjects [13]. Reciprocally depression has been observed with increased prevalence in patients suffering from chronic medical disorders. In addition to poor outcome and complicated disease course, the coexistence of CHF, diabetes and depression is associated with an increased burden of healthcare cost [13-15]. Hypertension has been believed to be the most frequent and conventional risk factor linked to CHF [16]. But we found a lower proportion of MDD patients with comorbid diabetes and hypertension, also the inpatient length of stay and overall cost per admission was lower compared to other comorbidities in depressed patients admitted for CHF management. While evaluating the relation of hypertension in heart failure patients with MDD, studies conducted in the past suggest depression as an independent predictor to put elderly hypertensive patients on the increased risk of developing heart failure [17].

Ranging from molecular and cellular levels to subsequent hemodynamic, thyroid hormone has its extensive impact on the cardiovascular system [18]. Studies have shown repercussions of thyroid dysfunction on cardiovascular dynamics and suggest an increased risk of CHF in hypothyroidism [19]. Hypothyroidism has been suggested to be strongly linked to depression and CHF concurrently [20]. Also, in our study, we found that a higher proportion of heart failure patients with depression had comorbid hypothyroidism than the non-MDD group, and they were at four-fold higher risk of in-hospital mortality. These patients had longer mean hospitalization stay and cost for the heart failure management related to other comorbidities seen in depressed patients. Chronic obstructive pulmonary disease (COPD) in addition to being prevalent in CHF, independently poses an increased risk of cardiovascular mortality and morbidity [21]. The association of COPD with depression has been frequently reported mainly accounted for by its chronicity. Some of the studies suggest the highest association of depression with COPD compared to other chronic disorders [22]. Analysis of our study inpatient population also adds to the evidence with the significantly high percentage of chronic lung diseases in CHF patients with comorbid depression.

Depending on the nature of substance, miscellaneous acute and chronic consequences of cardiac toxicities, ranging from ischemia, arrhythmias to cardiac arrest have been observed with substance abuse [23]. However, there is not much data available on the outcomes and prevalence of comorbidity of depression and substance abuse in heart failure. Analysis of our results showed a higher percentage of CHF patients with depression had comorbid drug abuse and these patients had a shorter length of hospitalization, yet higher healthcare cost during heart failure management. However, we could not find a clinically significant increase in mortality with substance abuse in CHF patients with depression. Cardiovascular disorders like cardiomyopathy, hypertension and arrhythmias have been identified with alcohol use [23]. In our study, we could categorize only 7.4% of the heart failure and depressed patients with co-diagnosis of alcohol abuse. But, alcohol abuse increases the risk of in-hospital mortality in depressed CHF patients by three times.

A study was done to evaluate the impact of MDD in hospitalized patients with Parkinson's disease [24], and as the goal of our study was to evaluate the outcomes in an inpatient sample, so we utilized the database that was used in that study. Data were extracted from the NIS to include the patients diagnosed with heart failure. We applied sampling weights given in the NIS [9] to generalize the estimates for the prevalence of medical and substance abuse comorbidities. The inpatient outcomes in terms of length of stay, cost and in-hospital mortality are generalizable to a bigger population than the sample studied using discharge weights. Using the NIS data set, there was a large sample size because we included 21,165 heart failure patients. This dataset is subject to negligible reporting bias, and all clinical and non-clinical information is coded independently of the individual physician, making it a theoretically more reliable source. This is the foremost study, to our knowledge, that reports the impact of various comorbidities in heart failure patients with MDD regarding hospital outcomes and in-hospital mortality. The limitation of using inpatient data (and not patient-level data) as the unit of analysis is that it does not translate to generalizability for all patients with heart failure. There may have been underreporting of chronic comorbidities in the NIS data because of the nature of an administrative database. We recommend that future research studies should examine the influence of comorbidities in a depressed patient with heart failure with clinical data.

## Conclusions

A middle-aged, white female with comorbid depression has a higher risk of hospitalization for heart failure. Medical comorbidities were less prevalent in CHF patients with depression. Hypothyroidism and drug abuse were prevalent in heart failure patients with MDD. Depressed CHF patients with comorbid hypothyroidism were hospitalized for a longer duration with higher inpatient cost and four times higher risk of mortality during hospitalization stay. Though alcohol abuse was associated with shorter hospital stay and cost, it increased the risk of in-hospital mortality by three times. Further studies are required to evaluate the underlying cause of worse hospital outcomes in depressed CHF patients with alcohol abuse and hypothyroidism. An integrated healthcare model is required for early diagnosis and treatment of depression and associated comorbidities in CHF patients to reduce mortality and improve post-CHF outcomes.

## References

[1] Gnanasekaran G (2011). Epidemiology of depression in heart failure. Heart Fail Clin.

[2] Rutledge T, Reis VA, Linke SE, Greenberg BH, Mills PJ (2006). Depression in heart failure a meta-analytic review of prevalence, intervention effects, and associations with clinical outcomes. J Am Coll Cardiol.

[3] Rustad JK, Stern TA, Hebert KA, Musselman DL (2013). Diagnosis and treatment of depression in patients with congestive heart failure: a review of the literature. Prim Care Companion CNS Disord.

[4] Sokoreli I, de Vries JJ, Pauws SC, Steyerberg EW (2016). Depression and anxiety as predictors of mortality among heart failure patients: systematic review and meta-analysis. Heart Fail Rev.

[5] Albert NM, Fonarow GC, Abraham WT (2009). Depression and clinical outcomes in heart failure: an OPTIMIZE-HF analysis. Am J Med.

[6] Freedland KE, Carney RM, Rich MW, Steinmeyer BC, Skala JA, Dávila-Román VG (2016). Depression and multiple rehospitalizations in patients with heart failure. Clin Cardiol.

[7] Adelborg K, Schmidt M, Sundboll J (2016). Mortality risk among heart failure patients with depression: a nationwide population-based cohort study. J Am Heart Assoc.

[8] Jackson CA, Manning WG Jr, Wells KB (1995). Impact of prior and current alcohol use on use of services by patients with depression and chronic medical illnesses. Health Serv Res.

[9] (2018). Overview of the national (nationwide) inpatient sample (NIS). Healthcare Cost and Utilization Project (HCUP). . 2017 [cited.

[10] Moser DK, Dracup K, Evangelista LS (2010). Comparison of prevalence of symptoms of depression, anxiety, and hostility in elderly patients with heart failure, myocardial infarction, and a coronary artery bypass graft. Heart Lung.

[11] Silverberg DS, Wexler D, Iaina A, Steinbruch S, Wollman Y, Schwartz D (2006). Anemia, chronic renal disease and congestive heart failure—the cardio renal anemia syndrome: the need for cooperation between cardiologists and nephrologists. Int Urol Nephrol.

[12] Vulser H, Wiernik E, Hoertel N (2016). Association between depression and anemia in otherwise healthy adults. Acta Psychiatr Scand.

[13] Katon WJ (2011). Epidemiology and treatment of depression in patients with chronic medical illness. Dialogues Clin Neurosci.

[14] Katon W, Lin EH, Von Korff M (2010). Integrating depression and chronic disease care among patients with diabetes and/or coronary heart disease: the design of the TEAMcare study. Contemp Clin Trials.

[15] Unützer J, Schoenbaum M, Katon WJ, Fan MY, Pincus HA, Hogan D, Taylor J (2009). Healthcare costs associated with depression in medically Ill fee-for-service medicare participants. J Am Geriatr Soc.

[16] Levy D, Larson MG, Vasan RS, Kannel WB, Ho KK (1996). The progression from hypertension to congestive heart failure. JAMA.

[17] Abramson J, Berger A, Krumholz HM, Vaccarino V (2001). Depression and risk of heart failure among older persons with isolated systolic hypertension. Arch Intern Med.

[18] Ascheim DD, Hryniewicz K (2002). Thyroid hormone metabolism in patients with congestive heart failure: the low triiodothyronine state. Thyroid.

[19] Stathatos N, Wartofsky L (2003). Perioperative management of patients with hypothyroidism. Endocrinol Metab Clin North Am.

[20] Bunevicius R, Varoneckas G, Prange AJ Jr, Hinderliter AL, Gintauskiene V, Girdler SS (2006). Depression and thyroid axis function in coronary artery disease: impact of cardiac impairment and gender. Clin Cardiol.

[21] Le Jemtel TH, Padeletti M, Jelic S (2007). Diagnostic and therapeutic challenges in patients with coexistent chronic obstructive pulmonary disease and chronic heart failure. J Am Coll Cardiol.

[22] Schane RE, Walter LC, Dinno A, Covinsky KE, Woodruff PG (2008). Prevalence and risk factors for depressive symptoms in persons with chronic obstructive pulmonary disease. J Gen Intern Med.

[23] Frishman WH, Del Vecchio A, Sanal S, Ismail A (2003). Cardiovascular manifestations of substance abuse: part 2: alcohol, amphetamines, heroin, cannabis, and caffeine. Heart Dis.

[24] Patel RS, Makani R, Mansuri Z, Patel U, Desai R, Chopra A (2017). Impact of depression on hospitalization and related outcomes for Parkinson's disease patients: a nationwide inpatient sample-based retrospective study. Cureus.

